# Kalamazoo Valley, (Mich.,) Medical Association

**Published:** 1867

**Authors:** 


### KALAMAZOO VALLEY, MICH., MEDICAL ASSOCIATION.

The Kalamazoo Valley, Mich., Medical Association convened its regular quarterly meeting Thursday.

Dr. S. S. French, of Battle Creek, President.

Several valuable papers were read.

Dr. Hitchcock offered a series of resolutions on criminal abortion, which were unanimously adopted, and a committee appointed to report at the next meeting, consisting of Drs. Alfred, Hitchcock, and Clapham.

The Association appointed the following delegates to the American Medical Association, which meets at Cincinnati, May 7th, 1867:—Drs. B. Barnum, Schoolcraft; Wm. Mottram and H. O. Hitchcock, Kalamazoo; and J. Andrews, Paw Paw.

The delegates were instructed to select substitutes, in case they should not be able to attend.

